# Evaluating Whether Melatonin Impacts Postoperative Sleep Following Total Joint Arthroplasty: A Systematic Review and Meta-analysis of Randomized Controlled Trials

**DOI:** 10.7759/cureus.107734

**Published:** 2026-04-26

**Authors:** Daniel I Razick, Muzammil Akhtar, Jimmy Wen, Gabriella O'Brien, Megan Kou, Adam A Razick, Nuraz M Syed, Sophia A Razick, Fayez S Siddiqui, Zachary C Lum

**Affiliations:** 1 Surgery, California Northstate University College of Medicine, Elk Grove, USA; 2 Physical Medicine and Rehabilitation, California Northstate University College of Medicine, Elk Grove, USA; 3 Orthopedics, Loyola Marymount University, Los Angeles, USA; 4 College of Medicine, California Northstate University College of Medicine, Elk Grove, USA; 5 Psychology, University of California Los Angeles, Los Angeles, USA; 6 Psychology, University of Texas at Austin, Austin, USA; 7 Psychology, University of California San Diego, San Diego, USA; 8 Internal Medicine, California Northstate University, Elk Grove, USA; 9 Orthopedic Surgery, University of California Davis Health, Sacramento, USA

**Keywords:** melatonin, sleep aid, total hip arthroplasty, total joint arthroplasty, total knee arthroplasty

## Abstract

Melatonin is a commonly used sleep aid, though its effects on postoperative sleep after total joint arthroplasty (TJA) remain unclear. Therefore, the objective of this study was to evaluate the effect of melatonin on subjective and objective sleep measures of postoperative sleep after TJA. The primary outcome of interest was the Pittsburgh Sleep Quality Index (PSQI). A search was performed utilizing the Boolean search phrase “(melatonin OR ramelteon OR tasimelteon OR agomelatine) AND (joint OR hip OR knee) AND (replacement OR arthroplasty).” Sleep duration, nightly awakenings, and patient-reported sleep outcomes were recorded when available. PSQI scores at six weeks were compared with a random-effects proportion meta-analysis weighted for individual study size. Across the 4 included randomized controlled trials, 455 patients were identified, of whom 227 received melatonin, and 228 received a placebo. Melatonin dosage was 5 mg in three studies and 6 mg in one. Across the four studies, melatonin did not significantly impact subjective or objective sleep outcomes. Meta-analysis of 6-week postoperative PSQI scores of 144 patients demonstrated no significant differences compared to 146 control patients (P = 0.74). Melatonin does not appear to have a significant impact on postoperative sleep following TJA. Further investigation is warranted to address other factors, such as pain and environmental disturbances, to improve sleep quality.

## Introduction and background

Melatonin is a hormone produced by the pineal gland and plays a prominent role in the sleep-wake cycle. By attenuating the wake-promoting signal of the circadian clock, melatonin promotes sleep and can be used to treat primary and secondary sleep disorders [1]. As of 2018, 2.1% of Americans reported using melatonin as a sleep aid, an increase from 0.4% in 2000 [2]. Given its growing use among the general population, its efficacy in improving sleep quality for patients in the postoperative period has been investigated [3]. Typical adult dosing in the United States is between 0.1 and 10 milligrams (mg) [4].

Within the field of total joint arthroplasty (TJA), the effects of TJA on sleep in the immediate postoperative period remain unclear [5]. Nithagon et al. report that sleep quality after TJA remains poor, despite growing evidence in the literature suggesting TJA improves sleep in the long term [5,6]. Factors that may contribute to poor postoperative sleep include pain, preoperative comorbidity, anesthesia, environmental stress, and severity of surgical trauma [7]. Given the importance of sleep in the postoperative period and the percentage of patients who experience poor sleep, it is critical to identify potential sleep modifiers such as sleep aids. A recent systematic review analyzing various sleep aids found zolpidem, fascia iliaca compartment block with dexmedetomidine, and perioperative methylprednisolone to improve sleep quality [6]. While the previous systematic review briefly discussed melatonin, the effect of exogenous melatonin remains unclear. Therefore, the primary purpose of this systematic review and meta-analysis was to evaluate the impact of melatonin on postoperative sleep in patients who underwent TJA, through subjective and objective sleep measures, analyzing the most recent studies. The primary outcome of interest was the Pittsburgh Sleep Quality Index (PSQI), a validated, patient-reported outcome measure assessing sleep duration, latency, and disturbances, with higher scores indicating poorer sleep quality [6].

This article was previously presented as a meeting abstract at the 2025 California Northstate University Annual Research Symposium on March 22, 2025.

## Review

Methods

Search Strategy

A search following guidelines established by the Preferred Reporting Items for Systematic Reviews and Meta-analyses (PRISMA) was performed in three databases on May 13, 2025: PubMed, Embase, and Cochrane Library. The query was performed utilizing Boolean search phrases. For PubMed, the phrase was “(melatonin [Mesh] OR ramelteon OR tasimelteon OR agomelatine) AND (joint OR hip OR knee) AND (replacement OR arthroplasty).” For Embase, the phrase was "('melatonin'/exp OR melatonin OR ramelteon OR tasimelteon OR agomelatine) AND ('arthroplasty'/exp OR arthroplasty OR 'joint replacement' OR hip OR knee)." For Cochrane Library, the phrase was "(melatonin OR ramelteon OR tasimelteon OR agomelatine) AND (arthroplasty OR "joint replacement" OR hip OR knee)." There were no restrictions set on the search. Studies were only included if they reported outcomes of randomized controlled trials (RCTs) involving the use of melatonin in patients who underwent TJA. Exclusion criteria included case reports, review articles, conference abstracts, studies performed on animals, pediatric studies, cadaveric/anatomical studies, expert opinions, and letters to the editor. This study was registered on PROSPERO (ID: CRD420250653194).

Study Selection

Two independent reviewers evaluated studies from the initial database search for eligibility criteria. A third senior author was available for any disputes. All included articles underwent rigorous reference search to determine whether additional studies could be added to the systematic review.

Data Extraction

Study variables extracted from each article included authors, details on study methodology, study period, number of patients, patient age, follow-up period, procedure type, melatonin dosage, and sleep measures. Inclusion and exclusion criteria were noted for all included studies. All studies excluded patients with recent use of sleep aids or conditions and medication use in which melatonin was contraindicated. Sleep measures included pre- and postoperative Pittsburgh Sleep Quality Index (PSQI) scores, Epworth Sleepiness Score, hours slept, Patient-Reported Outcomes Measurement Information System (PROMIS) sleep disturbance scores, and sleep efficiency. All extracted data were compiled for analysis using Microsoft Word (Microsoft Office 2011; Microsoft, Redmond, WA). Data was extracted by two independent reviewers, and the data was cross-verified after extraction to ensure the accuracy of the data. 

Quality Assessment and Risk of Bias

The Cochrane Collaboration's Risk of Bias (RoB) tool was utilized to evaluate the quality and RoB of the included articles [8]. Two reviewers assessed each article based on seven criteria, including random sequence generation, allocation concealment, blinding of participants and personnel, blinding of outcome assessment, incomplete outcome data, selective outcome reporting, and other sources of bias. Any disagreements were resolved through thorough re-reviewing until a consensus was reached.

The risk of bias was found to be low for all four studies, although some domains demonstrated "some concerns." The Cochrane RoB breakdown can be found in Table 1.

**Table 1:** Risk of bias assessment with the Cochrane risk of bias tool ROB: risk of bias

Study	Sequence Generation	Allocation Concealment	Blinding of participants and personnel for all outcomes	Blinding of outcome assessors for all outcomes	Incomplete outcome data for all outcomes	Selective outcome reporting	Other sources of bias	Overall
Clarkson et al. [9]	Some concerns	Low ROB	Low ROB	Low ROB	Low ROB	Low ROB	Low ROB	Low ROB
Haider et al. [10]	Low ROB	Low ROB	Low ROB	Low ROB	Low ROB	Low ROB	Some concerns	Low ROB
Kirksey et al. [11]	Low ROB	Low ROB	Low ROB	Low ROB	Low ROB	Low ROB	Some concerns	Low ROB
Lebrun et al. [12]	Some concerns	Low ROB	Low ROB	Low ROB	Low ROB	Some concerns	Low ROB	Low ROB

Statistical Analysis

Descriptive statistics (mean, percentage, standard deviation, range, median) are reported in this review when applicable and when available. For studies in which data were available, a forest plot was generated to compare PSQI scores at 6 weeks for patients receiving melatonin versus a placebo. Statistical heterogeneity was determined using the I2 statistic. A random-effects model was utilized to account for expected clinical and methodological heterogeneity among included studies. Effect sizes were reported as mean differences (MD) and corresponding 95% confidence intervals (CI). All statistical analyses were done using Cochrane’s Reviewer Manager web application (RevMan; Computer program, version 5.4, The Cochrane Collaboration, 2020).

Article Selection Process

**Figure 1:**
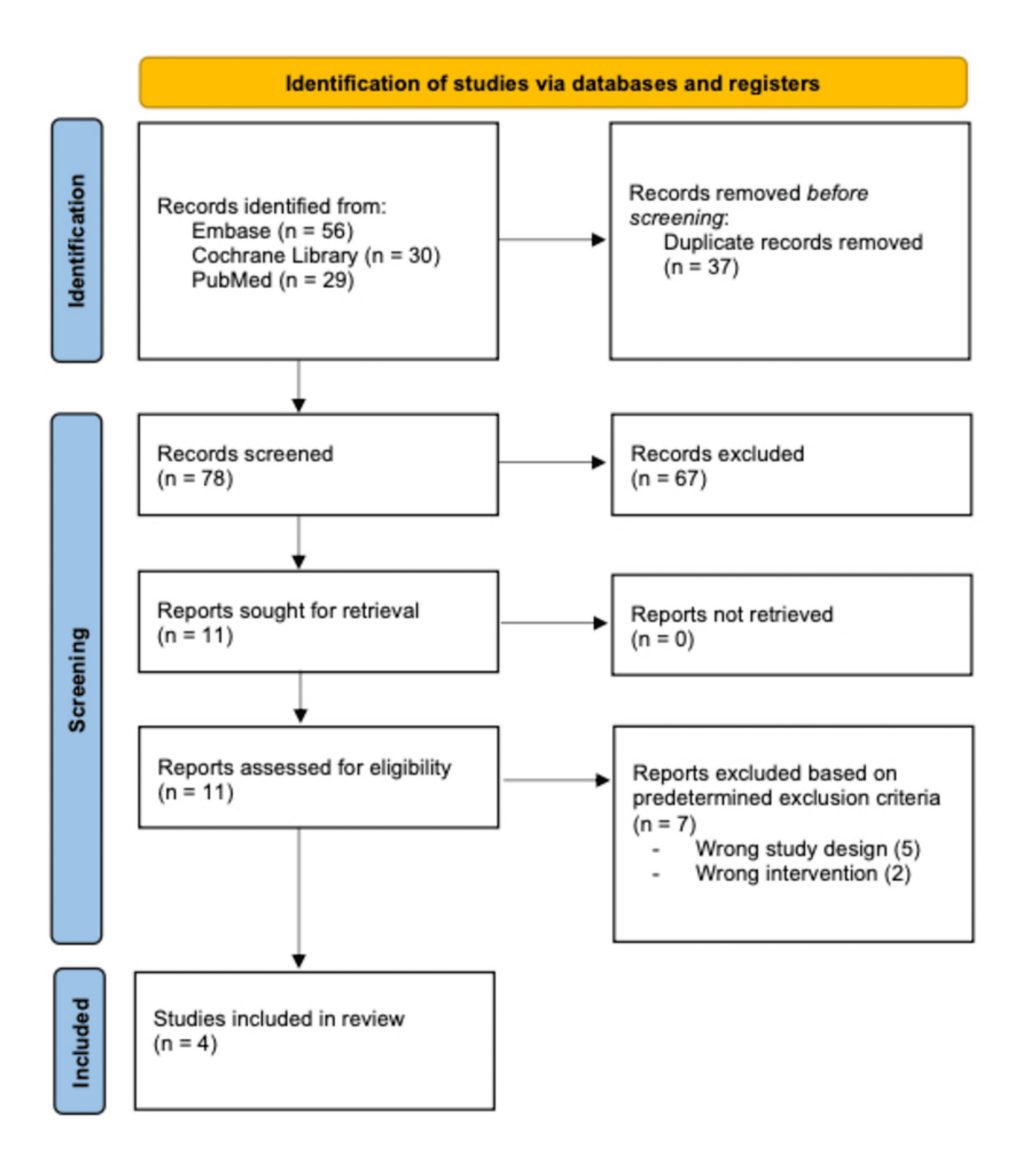
PRISMA flow diagram depicting the article selection process PRISMA: Preferred Reporting Items for Systematic reviews and Meta-Analyses

Upon the initial search of the PubMed, Embase, and Cochrane Library databases, 115 studies were identified, of which 37 duplicates were removed. The remaining 78 studies underwent full title and abstract review, of which 67 were removed based on our predetermined exclusion criteria. The remaining 11 studies underwent full-text review. Seven of these studies were excluded, as they did not fit our predetermined inclusion criteria, including incorrect study design (n = 5) and inappropriate intervention (n = 2). The remaining four were included in this systematic review, as depicted in the PRISMA flow diagram (Figure 1).

Results

Study Characteristics and Patient Demographic Information

The four studies included in this review were published between 2015 and 2024 [9-12]. All four studies were randomized controlled trials with a level of evidence (LOE) of I. Across the four studies, 455 patients were identified, of whom 227 received melatonin and 228 received a placebo. Mean ages for the experimental groups ranged from 64.6 to 70 and 63.4 to 67.8 in the control groups. Mean follow-up ranged from 3 days to 90 days. Melatonin dosage was 5 mg in three studies [10-12] and 6 mg in one study [9]. Table 2 summarizes the author of each study, study period, LOE, patient demographics, and melatonin dosage.

**Table 2:** Study Characteristics and Patient Demographics LOE: level of evidence; THA: total hip arthroplasty; TKA: total knee arthroplasty; mg: milligrams

Author	Study Period	LOE	Procedure Type	Patients (M/F)		Mean Age (years)		Mean Follow-up (days)	Melatonin Dosage
				Melatonin	Control	Melatonin	Control		
Clarkson et al. [9]	2018-2020	I	THA/TKA	58 (32/26)	60 (28/32)	64.6 ± 7.7	63.4 ± 8.2	42	6 mg
				23 THA/35 TKA	29 THA/31 TKA				
Haider et al. [10]	2021-2024	I	TKA	64 (23/41)	64 (16/48)	67 (41-83)	64 (32-84)	14	5 mg
Kirksey et al. [11]	2012-2013	I	TKA	19 (5/14)	18 (12/6)	70 ± 9.3	61.4 ± 14.3	3	5 mg
Lebrun et al. [12]	2023	I	TKA	86 (40/46)	86 (37/49)	66.8 ± 9.0	67.8 ± 8.4	90	5 mg

Sleep Outcomes

Outcomes regarding subjective and objective sleep measures were reported differently across the four studies and summarized in Table 3.

**Table 3:** Sleep outcomes PSQI: Pittsburgh Sleep Quality Index; POD: postoperative day; PROMIS SD: Patient-Reported Outcomes Measurement Information System Sleep Disturbance

Author	Sleep Outcomes					
Clarkson et al. [9]	Preoperative PSQI: Control: 6.8 ± 3.6; Melatonin: 6.8 ± 3.5 (p = 0.988)	PSQI (2 weeks): Control: 9.3 ± -4.6; Melatonin: 10.2 ± 4.2 (p = 0.309)			PSQI (6 weeks): Control: 8.7 ± -4.7; Melatonin: 8.8 ± -4.6 (p = 0.928)	
Haider et al. [10]	Average Epworth Sleepiness Score: Control: 6.2 ± 4.1; Melatonin: 7.2 ± 4.4 (p = 0.228)	Hours slept POD1-3: Control: 4.9 ± 2.0; Melatonin: 5.6 ± 1.8 (p = 0.073)		Hours slept POD4-6: Control: 5.7 ± 1.9; Melatonin: 6.0 ± 1.9 (p = 0.300)	Preoperative PROMIS SD: Control: 52.6 ± 7.8; Melatonin: 52.6 ± 7.9 (p = 0.974)	Postoperative PROMIS SD: Control: 56.6 ± 6.9; Melatonin: 57.2 ± 9.2 (p = 0.737)
Kirksey et al. [11]	Melatonin increased sleep efficiency by 4.4% (p = 0.150) and sleep time by 29 min (p = 0.067). No effect on subjective sleep quality.					
Lebrun et al. [12]	PSQI (6 weeks): Control: 10.5 ± 4.4; Melatonin 10.2 ± 4.2 (p = 0.66)		PSQI (90 days): Control: 7.5 ± 4.0; Melatonin: 8.1 ± 4.1 (p = 0.43)			

**Figure 2:**
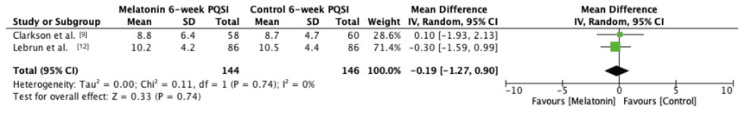
Six-week Pittsburgh Sleep Quality Index (PSQI) scores in the melatonin vs. control groups

Meta-analysis comparing six-week PSQI scores found no significant difference in scores between the melatonin and control groups (mean difference (MD) = -0.19, 95% CI: -1.27 to 0.90; P = 0.74). The I2 value was 0%, indicating no heterogeneity among the studies included. However, a random-effects model was used to account for potential clinical and methodological variability. The meta-analysis forest plot is depicted in Figure 2. 

**Figure 3:**
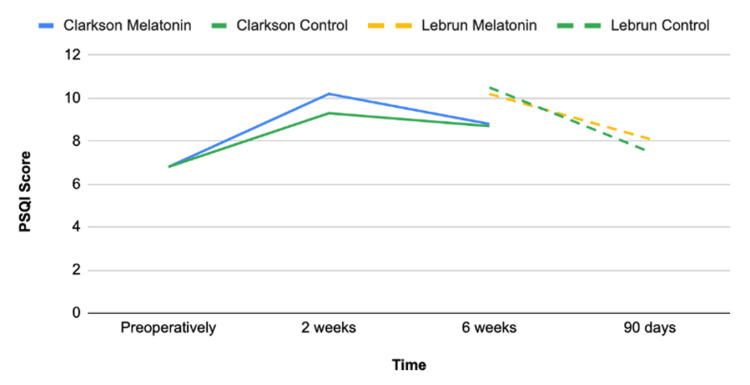
The effect of melatonin over time

The effects of melatonin over time are depicted in Figure 3.

Outcomes were categorized as subjective (e.g., PSQI, PROMIS, Epworth Sleepiness Score) and objective (e.g., sleep duration, sleep efficiency, nightly awakenings) measures to facilitate interpretation. 

Subjective Sleep Outcomes

Clarkson et al. was the only study to report outcomes on patients undergoing either total hip arthroplasty (THA) or total knee arthroplasty (TKA) and found no differences in postoperative sleep quality between control and melatonin groups. The study utilized the Pittsburgh Sleep Quality Index (PSQI) and found no significant differences at 2 weeks (P =0.309) or 6 weeks postoperatively (0.928). PSQI is graded on a scale of 0 to 21, with higher scores indicating worse sleep quality [9].

Haider et al. compared control and melatonin groups through the PROMIS Sleep Disturbance (PROMIS SD) scores and average Epworth Sleepiness Score. The PROMIS SD score increases (placebo: 4.0 ± 8.4, melatonin: 4.6 ± 8.2, P = .894), and sleep disturbance rates (placebo: 65%, melatonin: 65.4%) were similar for both groups [10]. 

Kirksey et al. reported that melatonin did not affect subjective sleep quality or daytime sleepiness [11]. 

Lebrun et al. also analyzed subjective sleep quality through PSQI and found that both control and melatonin groups demonstrated worse outcomes at 6 weeks compared to baseline (P <0.001). Scores returned to near preoperative baselines at 90 days. The study concluded that melatonin did not improve subjective sleep quality at 6 weeks or 90 days postoperatively (P = 0.66 and P = 0.43, respectively) [12].

Objective Sleep Outcomes

Haider et al. evaluated hours slept and nightly awakenings. The study found that melatonin patients trended toward more hours of sleep across postoperative days (POD) 1 to 3 (placebo: 4.9 ± 2.0, melatonin: 5.6 ± 1.8, P = .073), but no differences were observed on POD 4 or beyond. Patients in the melatonin group reported fewer night-time awakenings on POD 1, though this finding was not statistically significant (placebo: 4.4 ± 3.9, melatonin: 3.6 ± 2.4, P = .197) [10].

Kirksey et al. assessed sleep efficiency and sleep time. Sleep efficiency was non-significantly 4.4% greater in the melatonin group (P =0.150), and sleep time was longer by an average of 29 minutes (P = 0.067). The exact amount of sleep in each group was not reported [11].

Discussion

This systematic review and meta-analysis found no significant effect of melatonin on postoperative sleep outcomes after total joint arthroplasty. Across the four included RCTs, subjective and objective measures, including the PSQI, sleep duration, sleep efficiency, and nightly awakenings, showed no meaningful differences between placebo and melatonin groups at various postoperative time points. The overall findings in this systematic review suggest that melatonin does not significantly impact sleep following total joint arthroplasty. Given the limited number of RCTs available for inclusion, the present study should be interpreted as a focused systematic review with an exploratory meta-analysis rather than a definitive quantitative synthesis.

The impact of melatonin on postoperative sleep remains unclear, not only within orthopedic surgery but across various surgical specialties. A 2022 meta-analysis of RCTs evaluating the effect of melatonin on perioperative sleep in patients undergoing various procedures, including laparoscopic cholecystectomy, prostatectomy, knee arthroplasty, hip arthroplasty, and elective surgeries, concluded that melatonin can improve postoperative sleep [13]. However, it should be noted that the dosage included in the mentioned meta-analysis was 6 mg instead of 5 mg in the present meta-analysis. It is also worth mentioning that most of the surgeries performed were minimally invasive and required shorter postoperative recovery times. A 2022 RCT evaluating the effect of melatonin in orthopedic trauma patients found no significant improvement in sleep quality [14]. However, a 2024 RCT evaluating patients undergoing arthroscopic rotator cuff repair found improved PSQI scores and functional outcome scores in patients taking melatonin [15]. Given the results of the aforementioned studies, it can be postulated that the lack of sleep improvement with melatonin in TJA patients can be attributed to the aggressive nature of TJA and the physical toll patients undergo during the procedure, as compared to arthroscopic surgery. However, further direct comparisons are needed to understand the differences in sleep outcomes between the two types of orthopedic surgery.

Overall, the findings of the present study are consistent with prior systematic reviews suggesting melatonin alone may not significantly affect postoperative sleep. From a clinical perspective, the findings of the present study suggest that multimodal strategies addressing pain control and environmental factors, perhaps in combination with sleep aids, such as melatonin, may be more effective, particularly in physically demanding procedures such as TJA. 

While many factors contribute to patient outcomes, particularly in orthopedic surgery, the importance of sleep in the postoperative period should not be overlooked. Postoperative sleep disturbances are common and can have significant implications for recovery, as poor sleep quality has been associated with increased pain perception and promotes the development of a catabolic state [16]. Inadequate sleep can negatively impact patient satisfaction throughout the surgical experience, as it is associated with longer hospital stays and poorer cognitive function [17]. Exogenous melatonin is hypothesized to improve sleep by regulating circadian rhythms and mitigating sleep disruptions [1]. However, its lack of efficacy in the included studies suggests that other factors, such as environmental disturbances and pain, may play a more prominent role in decreasing sleep disturbances. Environmental factors within the hospital, such as noise, have been reported to heavily impact sleep. Deng et al. found that ward noise reduction improved early postoperative pain in patients undergoing plastic surgery, reduced psychological and physiological stresses, and improved sleep quality [18].

The relationship between pain and sleep is clear, with higher pain scores being associated with less sleep, and less sleep being associated with greater pain perception [19]. Addressing pain through a multimodal approach may lead to improved postoperative sleep outcomes. Given the limited impact of melatonin on postoperative sleep in TJA patients, future investigation should be targeted toward exploring alternative methods for optimizing sleep quality. Targeted light therapy and relaxation techniques, coupled with multimodal pain management, may provide insights into improving recovery outcomes [20]. Future research should also consider the influence of individual patient characteristics and comorbidities as they may impact postoperative sleep outcomes. Ultimately, a multifaceted approach to improving sleep quality in postoperative patients is vital to improving overall outcomes and warrants further investigation.

The findings of the present study should be considered in the context of its limitations. This study consisted of only four studies and comprised a small overall sample size, limiting its power to detect the effect of melatonin on sleep outcomes. As a result, no minimal clinically important difference (MCID) was found. There was significant heterogeneity in sleep outcome measures, as each study measured sleep through various subjective and objective metrics. PSQI at six weeks was the only overlapping measurement and was hence included in the meta-analysis. Follow-up periods were relatively short and varied widely across studies. Longer-term follow-up could reveal the full impact of melatonin on sleep outcomes. Melatonin dosage was not consistent across the four included studies. Most studies relied on subjective outcomes instead of objective sleep monitoring. Postoperative sleep is influenced by several factors, many of which were not controlled across studies, making it difficult to isolate the effects of melatonin on sleep quality. Moreover, important perioperative variables, including anesthesia techniques, recovery environment, and pain management protocols, were not consistently reported across the studies, limiting the authors' ability to assess the influence of these factors on postoperative sleep outcomes. Several included studies reported non-significant trends toward improved sleep outcomes in melatonin patients. However, these findings must be interpreted cautiously, as the small sample sizes may have limited statistical power, leading to an increased risk of type II errors. Finally, while publication bias exists, given the limited number of studies, there is a low risk of publication bias.

## Conclusions

Current evidence suggests that melatonin does not meaningfully improve postoperative sleep quality in patients undergoing TJA. Across the included studies, there were no significant differences between the melatonin (5-6 mg) and placebo groups in either subjective or objective sleep measures. Postoperative sleep in TJA patients is likely multifactorial and influenced by hospital environmental factors, such as noise, perioperative variables, such as anesthesia, and postoperative pain. Therefore, strategies aimed at improving pain control and optimizing the hospital environment by decreasing nighttime noise levels and interruptions may have a greater impact on sleep than melatonin alone. The findings of the present study are insufficient to establish a dose-dependent benefit of melatonin or define an optimal dose, highlighting the need for future RCTs that evaluate a broader dose range and account for other perioperative variables such as anesthesia. Finally, given the invasiveness of TJA, further investigation into less invasive orthopedic procedures may help elucidate whether the lack of benefit observed in the present study is specific to TJA or reflects a broader limitation of melatonin in improving postoperative sleep.
